# Chimerism in Myeloid Malignancies following Stem Cell Transplantation Using FluBu4 with and without Busulfan Pharmacokinetics versus BuCy

**DOI:** 10.1155/2017/8690416

**Published:** 2017-11-08

**Authors:** Shatha Farhan, Michael Bazydlo, Klodiana Neme, Nancy Mikulandric, Edward Peres, Nalini Janakiraman

**Affiliations:** ^1^Stem Cell Transplant Program, Henry Ford Hospital, 2799 W. Grand Blvd, Detroit, MI 48202, USA; ^2^Division of Biostatistics, Henry Ford Hospital, 2799 W. Grand Blvd, Detroit, MI 48202, USA; ^3^Division of Pharmacy, Henry Ford Hospital, 2799 W. Grand Blvd, Detroit, MI 48202, USA

## Abstract

In the era of precision medicine, the impact of personalized dosing of busulfan is not clear. We undertook a retrospective analysis of 78 patients with myeloid malignancies who received fludarabine and busulfan (FluBu4) with or without measuring Bu pharmacokinetics (Bu PK) and those who received busulfan with cyclophosphamide (BuCy). Fifty-five patients received FluBu4, of whom 21 had Bu PK measured, and 23 patients received BuCy. Total donor cell chimerism showed that the percentage of patients maintaining 100% donor chimerism on day 100 was 66.7%, 38.2%, and 73.9% in the FluBu4 with PK, FluBu4 with no PK, and BuCy, respectively (*P* = .001). Patients who had decreasing donor chimerism by day 100 were 23.8%, 52.9%, and 26.1% in the FluBu4 with PK, FluBu4 with no PK, and BuCy, respectively (*P* = .04). Bu PK group had fewer patients with less than 95% donor chimerism on day 30, which was not statistically significant, 5% (FluBu4 PK), 31% (FluBu4 with no PK), and 21% (BuCy) (*P* = .18). Survival distributions were not statistically significant (*P* = .11). Thus, personalized drug dosing can impact donor chimerism in myeloid malignancies. This will need to be examined in larger retrospective multicenter studies and prospective clinical trials.

## 1. Introduction

Allogeneic stem cell transplant (SCT) which depends on chemotherapy and immunotherapy (graft versus leukemia effect) is the only potential curative treatment for most patients with acute myeloid leukemia (AML), myelodysplastic syndrome (MDS), and other myeloid malignancies. However, despite the advances in allogeneic SCT, disease relapse is still a major cause of death [1–4].

Chimerism analysis is an important tool to assess the origin of hematopoietic cells after SCT. Discrimination between donor and recipient cells allows evaluation for engraftment as well as detection of imminent graft rejection but its use as prognostic indicator for relapse is controversial [5]. Many methods have been used over the years to assess chimerism including cytogenetics, fluorescein in situ hybridization, and variable number of tandem repeats. However a major limitation of most of these techniques is that they are time consuming and without quantification. Most recently, the use of short tandem repeats with the use of fluorescent labeling of the primers and PCR resolution products allowed accurate quantification of the degree of mixed chimerism [6].

Reduced toxicity ablative conditioning regimens are increasingly used in SCT. Busulfan (Bu) has been used for many years as a component of conditioning before SCT and now is being used more and more especially with the intravenous formulation which leads to more predictable delivery and probably improved clinical outcomes compared with oral Bu [7–9]. However, even with intravenous administration, the exposure may vary 3- to 4-fold. Recently, personalized dosing of Bu using the patient-specific Bu clearance has been used by some transplant centers. Target exposure is reflected in the measurement area under the plasma concentration-time curve (AUC) or concentration at steady state [10]. However, its impact on early and late transplant outcomes is not clear.

To explore the impact of measuring busulfan pharmacokinetics (Bu PK) in conditioning regimens on early donor chimerism in myeloid malignancies, we undertook a retrospective analysis of patients with myeloid disorders who received 4 days of fludarabine and busulfan (FluBu4) with or without measuring Bu PK and busulfan and cyclophosphamide (BuCy) at our center in the last 10 years.

## 2. Materials and Methods

### 2.1. Patients

Patients who underwent their first allogeneic SCT for AML, MDS, or myeloproliferative neoplasms involving myeloablative conditioning with FluBu4 or BuCy at our center between 2005 and 2016 were included in this retrospective analysis. Informed consent was obtained from each patient per institutional guidelines. The institutional review board also reviewed and approved this retrospective analysis.

### 2.2. SCT Conditioning Regimen

All patients received 1 of 3 myeloablative conditioning regimens consisting of fludarabine 40 mg/m2 with Bu 3.2 mg/kg daily for 4 days with or without Bu PK dose adjustment or Bu 3.2 mg/kg for 4 days with cyclophosphamide 60 mg/kg for 2 days. The choice of regimen was at the discretion of treating physician.

### 2.3. Graft versus Host Disease (GVHD) Prophylaxis

Posttransplantation graft versus host disease (GVHD) prophylaxis consisted of methotrexate on days 1, 3, 6, and 11 and tacrolimus. Patients receiving unrelated donor transplants received antithymocyte globulin 4.5 mg/kg pretransplantation in divided doses.

### 2.4. Chimerism Analysis

Bone marrow donor-recipient total cell chimerism analysis was performed on day 30 and day 100 using a quantitative fluorescence-based short tandem repeat polymerase chain reaction with capillary electrophoresis for polymerase chain reaction product resolution. Data are presented as peaks, and the AUC represents the percentage of host-versus-donor hematopoiesis.

### 2.5. Supportive Care

All supportive care measures including prophylactic antibiotics and antifungals were utilized according to institutional protocols. Ursodeoxycholic acid was started with the initiation of conditioning regimen.

### 2.6. Statistical Methods

Baseline characteristics were summarized by transplant group. Continuous variables were summarized as the mean, standard deviation, and range. Categorical variables were summarized as frequency counts and percentages. Patient and transplantation characteristics were compared using Fisher's exact and chi-squared test for categorical variables and Mann-Whitney's test for continuous variables. For overall tests,* P* < .05 was used to indicate statistical significance. Engraftment was defined as achieving an absolute neutrophil count of 500/*μ*l for 3 consecutive days. Time of platelet engraftment was defined as the first of 3 consecutive days with a platelet count 20,000/*μ*l without transfusion support. Criteria for complete remission (CR) after transplant included absence of circulating blasts, less than 5% marrow blasts, lack of chromosomal abnormalities, and documented donor cell engraftment. Overall survival was defined as the time from SCT to the time of death or last contact. It was calculated using the Kaplan-Meier estimate.

## 3. Results

### 3.1. Patient and Transplant Characteristics

In this study, 78 patients were identified and included. Characteristics of the patients are summarized in Table 1. There were 50 males and 28 females with a median age of 59 years. Diagnoses included AML (*n* = 49), MDS (*n* = 19), and myeloproliferative neoplasms (*n* = 10). Thirty-four patients had a matched related donor, 32 had a matched unrelated donor, and 12 had a mismatched unrelated donor SCT. Peripheral blood stem cells were used in all patients. Fifty-five patients received FluBu4, of whom 21 had Bu PK measured. BuCy was given in 23 patients. Bu dose was adjusted to more than 10% change based on PK in 81% of patients in the FluBu4 PK group. The change was more than 15% and more than 20% of the dose in 71% and 62% of patients in the FluBu4 PK group, respectively, median AUC targeted was 6000 uMolxMin, and median of actual target given was 5354 uMolxMin.

Gender, donor type, cytogenetics risk group, disease risk index, median blasts at time of SCT, CD34 dose, and antithymocyte globulin use were comparable (*P* > .30) between the 3 groups. The median CD34 dose was 3.9, 4.1, and 4.4 × 10^6^/kg of recipient in the FluBu4 with PK, FluBu4 with no PK, and BuCy, respectively. The median CD3 dose was 1.2, 1.2, and 1.1 × 10^8^/kg of recipient in the FluBu4 with PK, FluBu4 with no PK, and BuCy, respectively. Antithymocyte globulin was used in 57%, 62%, and 44% in the FluBu4 with PK, FluBu4 with no PK, and BuCy, respectively. Disease risk index was high or very high in 57%, 53%, and 60% of patients in the FluBu4 with PK, FluBu4 with no PK, and BuCy, respectively. Median blasts at time of transplant were 4% in all 3 groups. However, patients who received BuCy were younger with a median age of 45 years compared to patients who received FluBu4 with or without PK, 59 and 63 years, respectively (*P* < .001). In addition, patients who received FluBu4 with no PK had equal cases of MDS (44%) and AML (44%) as a diagnosis compared to FluBu4 with PK and BuCy who had more AML in those groups, 71% and 82%, respectively (*P* = .006).

### 3.2. Engraftment and Chimerism

Median time to neutrophil engraftment was 15, 12, and 14 days in the FluBu4 with PK, FluBu4 with no PK, and BuCy, respectively. Median time to platelet engraftment was 15, 13, and 14 days in the FluBu4 with PK, FluBu4 with no PK, and BuCy, respectively.

**Figure 1:**
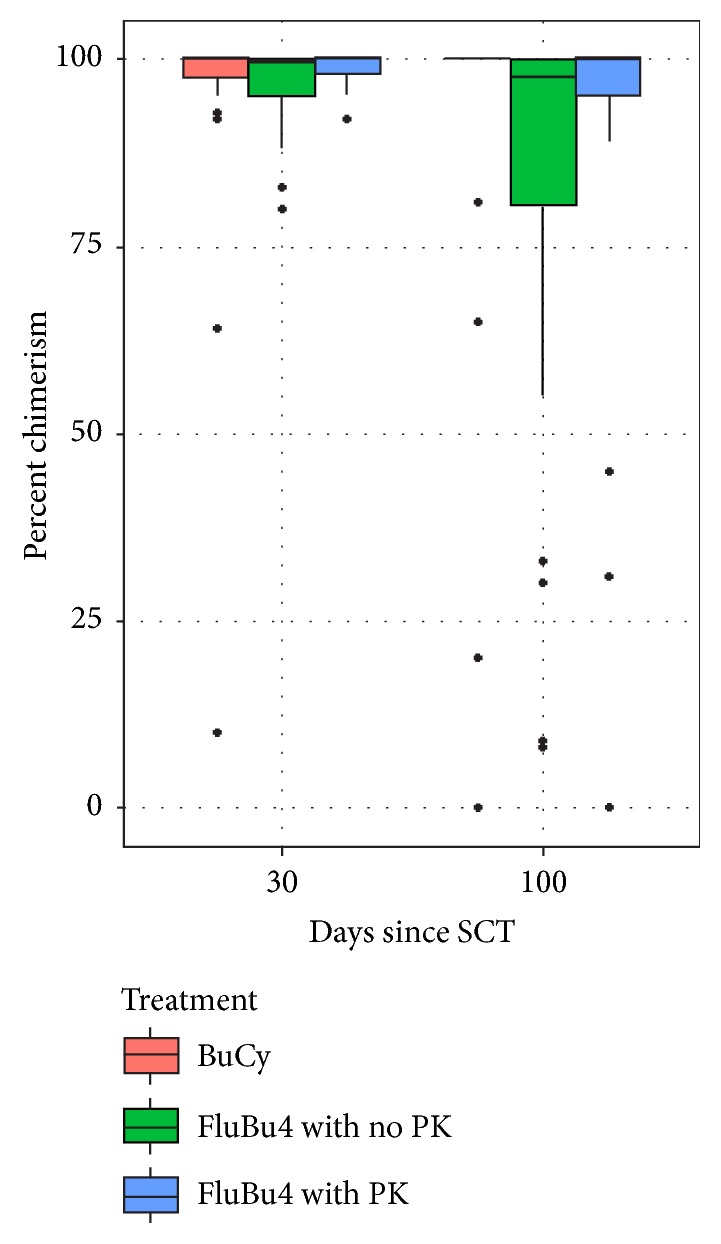
Boxplot for day 30 and day 100 chimerism for each of the 3 conditioning regimens. Bu, busulfan; Cy, cyclophosphamide; Flu, fludarabine; PK, pharmacokinetics; SCT, stem cell transplant.

We evaluated total donor cell chimerism values on days 30 and 100 after allogeneic SCT as a boxplot (Figure 1). The percentages of total donor chimerism which were grouped as 100%, 86%–99%, and less than 85% on day 30 and day 100 are summarized in Tables 2 and 3. Total donor cell chimerism analysis showed that the percentage of patients maintaining 100% donor chimerism on day 100 was 66.7 %, 38.2%, and 73.9% in the FluBu4 with PK, FluBu4 with no PK, and BuCy, respectively (*P* = .001). In addition, the percentage of patients who had decreasing total donor chimerism by day 100 was 23.8 %, 52.9%, and 26.1% in the FluBu4 with PK, FluBu4 with no PK, and BuCy, respectively (*P* = .04) (Table 4). The Bu PK group had fewer patients with less than 95% donor chimerism by day 30 compared to the other 2 groups (no PK and BuCy) although it was not statistically significant, 5% (FluBu4 PK), 31% (FluBu4 with no PK), and 21% (BuCy) (*P* = .18). Since patients who received BuCy were younger and the groups who got FluBu4 with PK and BuCy had more AML as above, we looked at multivariable analysis, which suggested that patients treated with FluBu4 without PK had higher odds of experiencing a decrease in chimerism from day 30 to day 100 than patients treated with BuCy or FluBu4 with PK after adjusting for age at transplant and disease. In addition, when we looked at the effect the treatment group has on being in the 100% chimerism at day 100 group versus being in the 86%–99% or less than 86% chimerism at day 100 group, adjusting for age and disease type as in Table 5, we found that the group of FluBu4 with no PK had a higher risk of not having 100% chimerism on day 100 with* P* = .066 (95% CI 0.06–1.09).

### 3.3. GVHD and Hepatic Venoocclusive Disease

None of the patients in the 3 groups developed hepatic venoocclusive disease. Grade II and grades III-IV acute GVHD were not different between the 3 groups (*P* = .13). The incidence of grade II acute GVHD was 19%, 17.6%, and 21.7% in the FluBu4 with PK, FluBu4 with no PK, and BuCy, respectively. Grades III-IV acute GVHD were 4.7%, 29%, and 17.4% in FluBu4 with PK, FluBu4 with no PK, and BuCy, respectively. Also looking at patients who had total donor chimerism more than 95% or less than 95% on day 100, rates of grade II and grades III-IV acute GVHD were not different between the 2 groups (*P* = .43). The incidence of grade II acute GVHD was 20% and 23% in the patients who had total donor chimerism more than 95% and less than 95% on day 100, respectively.

### 3.4. Relapse, Overall Survival, and Causes of Death

**Figure 2:**
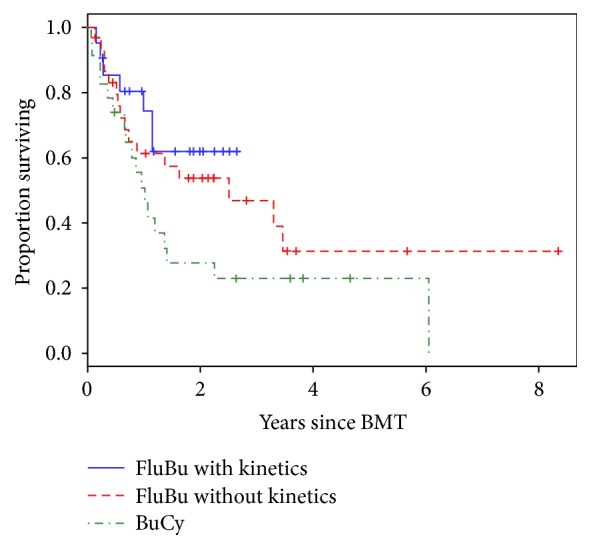
Kaplan-Meier curve for overall survival for allogeneic hematopoietic cell transplantation by conditioning regimen (*P* = .11). BMT, bone marrow transplant; Bu, busulfan; Cy, cyclophosphamide; Flu, fludarabine.

The median survival time for all patients was 1.40 years. Relapse rate was 24%, 47%, and 57% for FluBu4 with PK, FluBu4 with no PK, and BuCy, respectively (*P* = .085). When comparing the FluBu4 with no PK and BuCy groups, the odds ratio of relapse within the first 3 months was 2.23 (*P* = .194) for the no PK group. For the comparison of the FluBu4 with PK and BuCy groups, the odds ratio of relapse within the first 3 months was 1.12 (*P* = .87) for the FluBu4 with PK group. Survival distributions of the 3 treatment groups were not statistically significant (*P* = .11) (Figure 2). The cause of death was relapse in 28.5%, 35.3%, and 60% of patients in the FluBu4 with PK, FluBu4 with no PK, and BuCy groups, respectively, while the cause of death was GVHD in in 4.7%, 5.9%, and 8.7% patients in the FluBu4 with PK, FluBu4 with no PK, and BuCy, respectively. Sepsis and liver failure were cause of death in 8.7% of patients in the BuCy group. Cytomegalovirus antigenemia was documented in 35%, 34%, and 30% in the FluBu4 with PK, FluBu4 with no PK, and BuCy, respectively.

## 4. Discussion 

Myeloablative regimens, BuCy and FluBu4, remain the standard of care for patients undergoing SCT for AML or MDS if they can tolerate it as recently shown by the Blood and Marrow Transplant Clinical Trials Network (BMT CTN 0901) [11] phase III randomized trial comparing reduced intensity and myeloablative regimens in patients with AML or MDS. This shows that intensity of chemotherapy does matter in controlling aggressive myeloid disorders. However, not all myeloablative conditioning regimens are the same and they differ in toxicity and nonrelapse mortality.

Although the intravenous form of Bu bypasses the influence of gastrointestinal enzymes that affects the oral form, there is still a lot of inter- and intraindividual variability in Bu PK [12]. The practice guidelines committee of the American Society of Blood or Marrow Transplantation (ASBMT) sought to develop an evidence-based review of personalized dosing of Bu. However that was not feasible because of the lack of the necessary controlled studies and the published literature was too heterogeneous regarding patient population, conditioning regimen, Bu dosing, and Bu PK data [10]. Several studies provided recommendation of a maximally tolerated daily exposure of Bu of less than 6,000 mMxmin for 4 days [13], while others evaluated the maximally tolerated systemic exposure of intravenous Bu in combination with fludarabine and they showed that Bu can be safely escalated to an AUC of 7000 mMxmin or more without an appreciable difference in nonrelapse mortality [14, 15] although with need of more patients and longer follow-up in those studies. In our patients, the median AUC targeted was 6000 uMolxMin and the median actual target given was 5354 uMolxMin. Bu dose had to be adjusted based on PK in 81% of patients in the FluBu4 PK group, which is similar to the percentage reported by the Center for International Blood and Marrow Transplant Research (CIBMTR) where Bu dose was adjusted based on PK in 75% of patients who got FluBu4 [16].

Many studies were published trying to compare BuCy to FluBu4 [8, 16–19]. Most of these studies included heterogeneous group of patients with myeloid and lymphoid malignancies [17, 19]; not all of them used Bu PK [17–19] nor did they all look at chimerism kinetics [8, 16, 19]. Therefore, not a lot of the studies were looking at the early effect on chimerism especially in a homogeneous group of patients. In this study we looked at the effect of these 3 regimens, BuCy, FluBu4 with PK, and FluBu4 with no PK, on early donor chimerism in a homogenous group of patients with myeloid malignancies. We found that FluBu4 with PK and BuCy have a similar effect on early donor chimerism while in the FluBu4 with no PK group more patients had decreasing donor chimerism by day 100, which was statistically significant. Even when adjusted for disease and age at transplant, FluBu4 without PK had a higher risk of decreasing donor chimerism and more patients with less than 100% donor chimerism on day 100. On day 30, patients who had PK performed had lower rates of less than 95% donor chimerism compared to others, although not statistically significant. In a randomized trial done by Lee et al. [17], the BuFlu arm had a higher degree of recipient chimerism and a lower probability of complete chimerism at 4 weeks after SCT than the BuCy arm. In addition, they had worse survival in the BuFlu group, owing mainly to excessive disease relapse, which could be related to the lack of targeted dosing by PK. In another study by Liu et al. [18], patients were randomized to get BuCy or BuFlu; fludarabine was given after busulfan. All 106 evaluable patients achieved complete donor chimerism, defined in their study by more than 95% donor, by day +30 after transplantation. They also looked at chimerism on day +15 which was not statistically significant between the 2 groups but there was no mention of kinetic of chimerism after day 30. However, their patient population included only AML-CR1 with ages between 12 and 60 years, while our patient population has a group of higher risk diseases.

The MD Anderson Cancer Center group looked retrospectively at the effect of chimerism around day 100 (days +90 to +120) on the risk of relapse after SCT in patients with AML (81%) or MDS (19%) who got FluBu4 [20]. All patients received Bu 130 mg/m^2^ daily for 4 days or Bu given with PK dose adjustment, targeting a drug concentration AUC of 6000 mMxmin. Because of the high rates of full donor myeloid chimerism by days +90 to +120 in all patients they focused solely on the impact of T lymphocyte chimerism and concluded that early T lymphocyte chimerism testing is a useful approach for predicting AML/MDS disease recurrence in patients in CR1/CR2 at the time of transplantation. However, this study excluded patients who had disease progression before day +120. In addition, they looked at chimerism at one point of time without looking at changes in chimerism over time and without comparison of groups with and without PK. In our study we did not have data about lineage-specific chimerism because it is not done routinely for all the patients in our center; in addition that will create multiple small groups of patients which would not have the power to reflect and determine differences among those small groups. Koreth et al. [21] looked at 688 patients with hematologic malignancies who received FluBu to assess the impact of early donor chimerism on long-term outcomes. They showed that total donor chimerism independently predicts long-term relapse and survival and also concluded that total donor cell chimerism is sufficient and assessing T-subset chimerism is of no additional value to predicting these endpoints. Other studies that have suggested that such analyses cannot be used reliably [22–25] had either different diseases, smaller numbers of patients, or different condition regimens.

Regarding relapse, although there was high early 100% chimerism in the BuCy group, there was also high percentage of relapse in the same group. This is probably because that group had the longest follow-up. We started sending busulfan kinetics in our center in 2013. In the BuCy group some patients had follow-up (up to 3432 days) compared to FluBu4 with no PK (up to 2945 days) and the shortest follow-up was for FluBu4 with PK (up to 1373 days) which might explain more reported relapse in the BuCy group. In addition, mechanisms of early relapse are different than those of late relapse, as early relapse can be prevented by modifying the chemotherapy regimen or preemptive therapy with immunotherapy while late relapse might be related to other mechanisms like escape from the powerful cytotoxic effect of human leukocyte antigen-mismatched donor T cells [26, 27]. So when we looked at relapse in the first 3 months, there was a tendency for higher relapse in the FluBu4 with no PK compared to the other 2 groups, which was not statistically significant.

Regarding GVHD, there was no difference between the 3 groups in our study, similar to what was found by a meta-analysis by Ben-Barouch et al. In that analysis when they looked at the randomized trials only, the risk grade II–IV acute GVHD was similar between the 2 groups FluBu4 and BuCy [28].

Our study has limitations since it is a retrospective analysis, including potential selection bias and the presence of other confounding factors that were not measured like fludarabine kinetics and prior therapies. In addition because it is retrospective, we were unable to determine any causation, only association if any. Other limitations include the small number of patients, the short follow-up, and the absence of data on lineage-specific chimerism. The strengths of this study include looking at the kinetics of chimerism and not at just one point of time chimerism and a cohort of adult patients with only myeloid malignancies treated at a single center by the same physicians with uniform peripheral blood stem cell grafts and the widely used Bu based myeloablative preparative regimens with the standard-of-care methotrexate/tacrolimus-based with or without antithymocyte globulin GVHD prophylaxis.

## 5. Conclusion

Currently, there are no guidelines on how and when to perform personalized dosing of Bu as part of the conditioning regimen prior to SCT. Chimerism is one of the methods used to monitor disease after SCT. However, interpretation of the results and techniques is not yet standardized. In this small cohort, we found that patients with myeloid disorders who received FluBu4 with Bu PK had a trend for higher donor chimerism similar to BuCy on day 100, while FluBu4 with no PK had a tendency to lose donor chimerism by day 100 and had more patients with less than 95% donor chimerism by day 30. Thus, in the era of precision medicine, the conditioning regimens and personalized dosing may impact early donor chimerism. This is especially important in myeloid disorders. This will need to be examined in larger retrospective multicenter studies like CIBMTR and prospective clinical trials.

## Figures and Tables

**Table 1:** Summary of patient characteristics by treatment group.

	FluBu4 with PK	FluBu4 without PK	BuCy	*P* value
	*N* = 21	*N* = 34	*N* = 23	
	(% or range)	(% or range)	(% or range)	
Gender	M 14 (67%)	M 24 (70%)	M 12 (52%)	.349
	F 7 (33%)	F 10 (30%)	F 11 (48%)	
Median age at time of SCT	59 (41–70)	63 (48–72)	45 (22–63)	<.001
Disease				.006
Acute myeloid leukemia	15 (71%)	15 (44%)	19 (82%)	
MPN/MDS	6 (29%)	19 (56%)	4 (18%)	
Median blasts in bone marrow at time of SCT	4% (1–40%)	4% (1–20%)	4% (1–90%)	.910
Cytogenetic risk				.521
High	14 (67%)	17 (50%)	14 (61%)	
Intermediate	6 (29%)	16 (47%)	9 (39%)	
Low	1 (4%)	1 (3%)	0 (0%)	
Disease risk index				.372
Intermediate	9 (43%)	16 (47%)	7 (30%)	
High	5 (24%)	13 (38%)	9 (39%)	
Very high	7 (33%)	5 (15%)	7 (30%)	
Antithymocyte globulin use	12 (57%)	21 (62%)	10 (44%)	.38
CD34 dose × 10^6^	3.9 ± 1.5	4.1 ± 1.3	4.4 ± 2	.534
Donor type				.401
Matched related donor	9 (43%)	13 (38%)	12 (52%)	
Matched unrelated donor	7 (33%)	15 (44%)	10 (43%)	
Mismatched unrelated donor	5 (24%)	6 (18%)	1 (5%)	

Bu, busulfan; Cy, cyclophosphamide; F, female; Flu, fludarabine; M, male; MDS, myelodysplastic syndrome; MPN; myeloproliferative neoplasm; SCT, stem cell transplant.

**Table 2:** Total donor chimerism on day 30.

Chimerism results at day 30				*P* value
	FluBu4 with PK	FluBu4 with no PK	BuCy	
100% donor	14	17	16	.311
86%–99%	7	15	5	
<85%	0	2	2	

Bu, busulfan; Cy, cyclophosphamide; Flu, fludarabine; PK, pharmacokinetics.

**Table 3:** Total donor chimerism on day 100.

Chimerism results at day 100				*P* value
	FluBu4 with PK	FluBu4 with no PK	BuCy	
100% donor	14	13	17	.006
86%–99%	3	12	0	
<85%	4	9	6	

Bu, busulfan; Cy, cyclophosphamide; Flu, fludarabine; PK, pharmacokinetics.

**Table 4:** Decreasing and nondecreasing total chimerism between day 30 and day 100.

	Decreasing *N* (%)	Nondecreasing *N* (%)	*P* value
FluBu4 with no PK	18 (52.9)	16 (47.1)	.04
FluBu4 with PK	5 (23.8)	16 (76.2)	
BuCy	6 (26.1)	17 (73.9)	

Bu, busulfan; Cy, cyclophosphamide; Flu, fludarabine; PK, pharmacokinetics.

**Table 5:** Effect on the treatment group on being in the 100% chimerism on day 100 group versus being in the 86%–99% or <86% chimerism on day 100, adjusting for age and disease type.

Variable	OR	95% CI	*P* value
Treatment (versus BuCy)			
FluBu4 with kinetics	0.73	0.17–3.11	.672
FluBu4 without Kinetics	0.25	0.06–1.09	.066
Age at transplant	1.01	0.96–1.06	.790
Disease	1.53	0.57–4.13	.403

Bu, busulfan; Cy, cyclophosphamide; Flu, fludarabine; OR, odds ratio.
